# Posterior fossa decompression in syndromic children with chiari-like posterior fossa crowding: a nationwide US-based study

**DOI:** 10.1007/s00381-025-06954-7

**Published:** 2025-11-11

**Authors:** Victor Gabriel El-Hajj, Josué Aganze Mwambali, Ihab Ahmad Al-Rikabi, Erik Öhlen, Maria Gharios, Victor E. Staartjes, Joanna M. Roy, Basel Musmar, Pascal Jabbour, Erik Edström, Adrian Elmi-Terander

**Affiliations:** 1 Department of Clinical Neuroscience, Karolinska Institutet, Stockholm, Sweden; 2 Faculty of Medicine, Université Catholique de Bukavu, Bukavu, Democratic Republic of Congo; 3 Machine Intelligence in Clinical Neuroscience & Microsurgical Neuroanatomy (MICN) Laboratory, Department of Neurosurgery, University Hospital Zurich, Clinical Neuroscience Center, University of Zurich, Zurich, Switzerland; 4 Department of Neurosurgery, Thomas Jefferson University Hospital, Philadelphia, PA USA; 5 Capio Spine Center Stockholm, Löwenströmska Hospital, Upplands Väsby, Sweden; 6 Department of Medical Sciences, Örebro University, Örebro, Sweden

**Keywords:** Chiari malformation, Syndromic, Posterior fossa, Pediatric

## Abstract

**Introduction:**

Posterior fossa crowding, due to cerebellar tonsil herniation, often requires surgery with posterior fossa decompression (PFD). Although most cases are due to a Chiari-1 malformation (CM1), some are due to concomitant congenital conditions, mimicking a radiological CM1. The aim of this study was to compare PFD outcomes between CM1 and the syndromic Chiari-like crowding of the posterior fossa. A national pediatric surgical database was used to compare baseline characteristics and short-term postoperative outcomes.

**Methods:**

Pediatric patients undergoing PFD (2012–2021) were identified in the ACS NSQIP-P database. Baseline characteristics and 30-day outcomes were compared between syndromic and non-syndromic cases. Multivariate logistic regression identified factors associated with readmission and reoperation.

**Results:**

Among 6,910 patients who underwent PFD, 752 (11%) had associated syndromes or conditions. The most common syndromes were chondrodystrophies (28%), fetal alcohol syndrome (14%), and Klippel-Feil syndrome (10%). Syndromic patients were younger, had lower BMI, and exhibited a significantly higher prevalence of comorbidities, including pulmonary, gastrointestinal, cardiac, and neurological conditions (all *p* < 0.001). They also experienced longer hospital stays (mean 5.17 vs. 3.64 days; *p* = 0.010), higher rates of 30-day complications (12% vs. 8.7%; *p* = 0.003), readmissions (9.4% vs. 7.1%; *p* = 0.021), and reoperations (6.6% vs. 2.9%; *p* < 0.001). In adjusted analysis, syndromic cases were not independently associated with increased 30-day complications or readmissions but remained significantly associated with 30-day reoperation (OR 1.51; 95% CI 1.03–2.19; *p* = 0.032).

**Conclusion:**

The syndromic Chiari-like condition is associated with distinct baseline characteristics and higher short-term complication rates following PFD compared to true CM1. While syndromic status was independently associated with reoperation, it was not an independent predictor of overall complications or readmissions. These findings suggest that patients with syndromes mimicking CM1 represent a clinically and etiologically distinct entity, warranting tailored management and further investigation.

## Introduction

Chiari Malformation type 1 (CM1) is an abnormality of the hindbrain characterized by a structural defect in the base of the skull and cerebellum, which leads to herniation of the cerebellar tonsils [1, 2]. Its prevalence is estimated to be in the range of 1 per 100 to 1 per 5000 individuals with a female predilection [3] and almost 85% of cases being asymptomatic [2].

CM1 has been associated with several anatomical abnormalities that contribute to its clinical features [1, 4–8]. In the skull, common findings include basilar skull and craniovertebral junction anomalies, underdeveloped or shortened supraocciput, and smaller, shallower posterior fossa. Other abnormalities such as platybasia and basilar impression may also be noted.

The exact cause of CM1 remains unclear, with multiple theories proposed [6]. Some suggest a congenital anomaly leading to posterior fossa underdevelopment, while others point to altered CSF dynamics [9], connective tissue abnormalities [2, 5, 10], or mechanical traction from conditions like tethered cord syndrome [4, 6]. Additional hypotheses include intracranial hypertension, genetic predisposition, and skull or brain volume changes. Despite these insights, CM1’s etiology remains unresolved, requiring further research [6].

While the majority of cases of posterior fossa crowding are sporadic and lack genetic correlate, recent studies have implicated the role of genetics. Evidence of familial aggregation has been observed, including twin patients [11, 12]. Recent advances in genomic technologies have led to the identification of specific genes, involved in the advent of posterior fossa crowding, particularly in families with connective tissue disorders. These genes appear to be most active during developmental stages [13, 14]. Additionally, posterior fossa crowding has been linked to several genetic conditions such as Ehlers-Danlos syndrome, achondroplasia, Klippel-Feil syndrome, Hajdu-Cheney syndrome, familial hypophosphatemic rickets, further strengthening its genetic component [2, 8, 15].

The link between posterior fossa crowding and congenital conditions supports a genetic contribution, the implications for PFD in patients with concomitant congenital conditions remain largely unexplored. This study aims to analyze surgically treated cases with PFD from a large nationwide US-based database, focusing on whether (syndromic) posterior fossa crowding with concomitant congenital conditions or syndromes should be considered a distinct entity from true CM1. To this end, we examine differences in baseline characteristics and short-term postoperative outcomes available in the American College of Surgeons National Surgical Quality Improvement Program (ACS NSQIP)-Pediatric database.

## Methods

### Population

The American College of Surgeons National Surgical Quality Improvement Program-Pediatrics (ACS NSQIP-P) Database is a comprehensive and national US-based database that specifically focuses on quality improvement in the context of pediatric surgery. The database includes detailed and standardized data related to pediatric surgical procedures and conditions across a wide range of specialties, including neurosurgery.

The ACS NSQIP Pediatric Database includes multiple healthcare institutions and hospitals across the United States. It includes de-identified data on preoperative risk factors, intraoperative details, and 30-day postoperative outcomes for pediatric patients undergoing surgical procedures.

In this study, the NSQIP-P was queried from 2012 to 2021 to identify pediatric patients undergoing PFD procedures. The previously established Common Procedural Terminology (CPT) code 61343 was used to detect cases of PFD [16, 17]. All patients with simultaneous malignancies or those undergoing concomitant tumor or vascular malformation surgeries were excluded, leaving patients undergoing PFD for posterior fossa crowding. Patients with concomitant myelomeningocele were considered to have a CM type 2 (CM2) and were excluded from the analysis.

### Variables and primary outcomes

Baseline data included age, sex, BMI, race, ethnicity, American Society of Anesthesiologists (ASA) classification, and other comorbidities, genetic, or congenital conditions from the ACS NSQIP-P. Congenital or genetic anomalies were either specified with a clear diagnosis or unspecified. In the latter, the patient was born with an unspecified congenital anomaly including craniofacial (ex: cleft lip or palate), or internal organ defects (ex: anal atresia or abdominal wall defects). The presence of myelomeningocele, syringomyelia, hydrocephalus was extracted through the available ICD codes.

The population was divided in two cohorts depending on the syndromic status.

Outcome data included length of hospital stay, 30-day complications, discharge disposition, mortality, readmission, and reoperation. The unplanned 30-day readmission and 30-day reoperation were the primary study outcomes. Surgical cases with missing data for these outcomes were excluded from the analysis.

### Statistical analysis

Descriptive statistics were used to define the overall study cohort.

The Mann–Whitney U and Chi-square tests were used to compare continuous and categorical variables between groups. Statistically significant variables in the univariable analysis, as well as those clinically relevant were included in multivariable logistic regression.

A *p* ≤ 0.05 was set as the threshold for statistical significance for two-sided tests. Analyses were completed using R (version 4.2.1, The R Foundation for Statistical Computing).

## Results

### Syndromes associated with posterior fossa crowding

A total of 6,910 patients undergoing PFD were included in the study, of which 752 (11%) had associated syndromes or conditions. Among these, 409 patients (54%) had specific, identified syndromes or conditions. The most common were chondrodystrophies, primarily achondroplasia (28%), Fetal Alcohol syndrome (14%), Klippel-Feil syndrome (10%), neurofibromatosis (10%), and Ehler-Danlos syndrome (7%). Less common syndromes included: Pituitary dwarfism (5%), craniosynostosis (4.3%), cerebellar hypoplasia (3.4%), Down’s syndrome, and velocardiofacial syndrome, (2.4% each), osteogenesis imperfecta (2.2%), and mucopolysaccharidoses (2.2%) (Table 1). The remaining 343 patients (46%) had verified but unidentified syndromes or conditions. Among this group, the most frequently reported features were skull and facial bone defects, observed in 65% of cases, and internal organ defects, present in 24%. Great vessel anomalies were reported in 1.2% of cases, while the exact features of the syndrome or condition remained unknown in 9.6% of patients.

**Table 1:** Included syndromes and conditions associated with posterior fossa crowding in our cohort

Total number of patients included	*N* = 6,910
**Patients with verified syndromes or conditions**	**752 (11%)**
**Identified syndromes and conditions**	**409 (54%)**
Chondrodystrophies (most commonly achondroplasia)	116 (28%)
Fetal Alcohol Syndrome	59 (14%)
Klippel-Feil syndrome	42 (10%)
Neurofibromatosis	42 (10%)
Ehler Danlos syndrome	29 (7.0%)
Pituitary dwarfism	21 (5.0%)
Craniosynostosis	18 (4.3%)
Cerebellar hypoplasia	14 (3.4%)
Down's syndrome (trisomy 21)	10 (2.4%)
Velocardiofacial syndrome (22q11.2 deletion)	10 (2.4%)
Osteogenesis imperfecta	9 (2.2%)
Mucopolysaccharidoses	9 (2.2%)
Cystic fibrosis	8 (1.9%)
Inborn errors of metabolism	7 (1.7%)
Mitochondrial diseases	5 (1.2%)
Muscular dystrophies	4 (1.0%)
Prader-Willi syndrome	2 (0.5%)
Klinefelter's syndrome	1 (0.2%)
Marfan's syndrome	1 (0.2%)
Angelman syndrome	1 (0.2%)
Edward's syndrome (trisomy 18)	1 (0.2%)
**Features in unidentified but verified syndromes or condition**	**343 (46%)**
Skull and facial bones defects	223 (65%)
Internal organ defects	84 (24%)
Great vessel anomaly	4 (1.2%)
Unknown	32 (9.6%)

### Baseline patient characteristics and comorbidities

Baseline characteristics of all patients undergoing PFD, were compared (Table 2). The group with associated syndromes was significantly younger, with a mean age of 7.08 (SD 5.13) years compared to 9.75 (SD 5.15) years in the group without associated syndromes (*p* < 0.001). Additionally, the proportion of males was higher in the group with associated syndromes (50% vs. 43%; *p* < 0.001). The group with associated syndromes also had a lower mean BMI as compared to the group without associated syndromes (19.50 vs. 20.77; *p* < 0.001). A higher proportion of patients with associated syndromes were born prematurely (18% vs. 12%; *p* < 0.001).

**Table 2:** Baseline characteristics in patients with versus without associated syndromes, undergoing posterior fossa decompression surgery

Variable	Syndromic, *N* = 752	Non-syndromic, *N* = 6,158	*p*-value
**Male sex**	375 (50%)	2,670 (43%)	** < 0.001**
**Age**	7.08 (5.13)	9.75 (5.15)	** < 0.001**
**BMI**	19.50 (6.00)	20.77 (7.25)	** < 0.001**
**Race**			0.66
Asian	16 (2.1%)	97 (1.6%)	
Black or African American	56 (7.4%)	480 (7.8%)	
Other	6 (0.8%)	40 (0.6%)	
White	674 (90%)	5,541 (90%)	
**Admitted from**			**0.003**
Home/clinic/doctor’s office	711 (95%)	5,969 (97%)	
Emergency room	39 (5.2%)	177 (2.9%)	
Other	2 (0.3%)	12 (0.2%)	
**Premature birth**	129 (18%)	676 (12%)	** < 0.001**
Missing	53	715	
**Ventilator dependent**	38 (5.1%)	63 (1.0%)	** < 0.001**
**Asthma**	104 (14%)	540 (8.8%)	** < 0.001**
**Oxygen support**	53 (7.0%)	61 (1.0%)	** < 0.001**
**Tracheostomy**	33 (4.4%)	18 (0.3%)	** < 0.001**
**Structural pulmonary abnormality**	197 (26%)	378 (6.1%)	** < 0.001**
**Gastrointestinal condition**	152 (20%)	450 (7.3%)	** < 0.001**
**Previous cardiac surgery**	53 (7.0%)	110 (1.8%)	** < 0.001**
**Cardiac risk factors**			** < 0.001**
Major cardiac risk factors	76 (10%)	148 (2.4%)	
Minor cardiac risk factors	79 (11%)	202 (3.3%)	
None	597 (79%)	5,808 (94%)	
**Developmental delay/Impaired cognitive status**	344 (46%)	1,050 (17%)	** < 0.001**
**Seizure disorder**	57 (7.6%)	444 (7.2%)	0.71
**Cerebral palsy**	22 (2.9%)	71 (1.2%)	** < 0.001**
**Neuromuscular disorder**	116 (15%)	327 (5.3%)	** < 0.001**
**Intraventricular hemorrhage**			0.71
Grade 1	0 (0%)	6 (< 0.1%)	
Grade 2	1 (0.1%)	2 (< 0.1%)	
Grade 3	1 (0.1%)	10 (0.2%)	
Grade 4	1 (0.1%)	10 (0.2%)	
IVH reported but no grade assigned	2 (0.3%)	18 (0.3%)	
No IVH reported	747 (99%)	6,112 (99%)	
**Steroid use**	29 (3.9%)	97 (1.6%)	** < 0.001**
**Ostomy**	76 (10%)	102 (1.7%)	** < 0.001**
**Nutritional support**	90 (12%)	109 (1.8%)	** < 0.001**
**Hematologic disorder**	31 (4.1%)	126 (2.0%)	** < 0.001**
**Past history of cancer**	6 (0.8%)	22 (0.4%)	0.12
**Hydrocephalus**	76 (10%)	260 (4.2%)	** < 0.001**
**Central apnea**	28 (3.7%)	42 (0.7%)	** < 0.001**
**Syringomyelia**	18 (2.4%)	232 (3.8%)	0.057
**Scoliosis**	7 (0.9%)	44 (0.7%)	0.51
**Tethered cord**	12 (1.6%)	56 (0.9%)	0.072

Syndromic patients had significantly higher rates of pulmonary, gastrointestinal, cardiac, neuromuscular, and developmental comorbidities, as well as greater need for supportive care, compared to those non-syndromic patients (*p* < 0.05).

Hydrocephalus was significantly more common in the syndromic group, affecting 10% (*n* = 76) of patients, compared to 4.2% (*n* = 260) in the non-syndromic group (*p* < 0.001). Central apnea was also more prevalent in the syndromic group, with 3.7% (*n* = 28) of patients affected, compared to 0.7% (*n* = 42) in the non-syndromic group (*p* < 0.001). For syringomyelia (3.8% vs. 2.4%; *p* = 0.057), scoliosis (0.7% vs. 0.9%; *p* = 0.51), and tethered cord (0.9% vs. 1.6%; *p* = 0.072), the differences between groups were not statistically significant.

### Surgical characteristics

In terms of surgical characteristics (Table 3), the majority of both groups underwent elective surgery, with no significant difference among groups (*p* = 0.057).

**Table 3:** Surgical characteristics in patients with versus without associated syndromes undergoing posterior fossa decompression

Variable	Syndromic, *N* = 752	Non-syndromic, *N* = 6,158	*p*-value
**Admission**			0.057
Elective	726 (97%)	6,015 (98%)	
Urgent	26 (3.5%)	143 (2.3%)	
**American Society of Anesthesiologists (ASA) Class**			** < 0.001**
1	2 (0.3%)	37 (0.6%)	
2	167 (22%)	4,170 (68%)	
3	571 (76%)	1,945 (32%)	
4	12 (1.6%)	6 (< 0.1%)	
**Operation duration (mins)**	162 (80)	153 (65)	0.12
**CSF diversion procedure**	28 (3.7%)	82 (1.3%)	** < 0.001**
**Arthrodesis**	40 (5.3%)	57 (0.9%)	** < 0.001**

Regarding the American Society of Anesthesiologists (ASA) class, a significant difference was observed between the two groups. In the syndromic group, 76% (*n* = 571) of patients were classified as ASA Class 3 and 22% (*n* = 167) as ASA Class 2, compared to 32% (*n* = 1,945) ASA Class 3 and 68% (*n* = 4,170) ASA Class 2 in the non-syndromic group (*p* < 0.001). This indicates that patients in the syndromic group had significantly higher comorbidity levels.

There was no difference in the average duration of surgery between groups (162 vs. 153 min; *p* = 0.12). In addition, patients in the syndromic group were more likely to undergo cerebrospinal fluid (CSF) diversion procedures as compared to those in the non-syndromic group (3.7% vs. 1.3%; *p* < 0.001). Similarly, arthrodesis was performed more frequently in the syndromic group (5.3%, *n* = 40) compared to 0.9% (*n* = 57) in the non-syndromic group (*p* < 0.001).

### Surgical outcomes

In terms of postoperative outcomes (Table 4), syndromic patients experienced a significantly higher incidence of any 30-day complication (8.7%, *n* = 536 vs. 12%, *n* = 90; *p* = 0.003). Surgical site infections (3.6%, *n* = 27 vs. 2.3%, *n* = 144, *p* = 0.049), postoperative sepsis (1.2%, *n* = 9 vs. 0.4%, n = 24, *p* = 0.007), CSF leaks (1.3%, *n* = 10 vs. 0.5% *n* = 31, *p* = 0.011).

**Table 4:** Posterior fossa decompression outcomes in patients with versus without associated syndromes

Variable	Syndromic, *N* = 752	Non-syndromic, *N* = 6,158	*p*-value
**Any 30-day complication**	90 (12%)	536 (8.7%)	**0.003**
**Surgical site infection**	27 (3.6%)	144 (2.3%)	**0.049**
**Wound dehiscence**	9 (1.2%)	71 (1.2%)	0.92
**Meningitis**	9 (1.2%)	59 (1.0%)	0.53
**CSF leak**	10 (1.3%)	31 (0.5%)	**0.011**
**Postoperative sepsis**	9 (1.2%)	24 (0.4%)	**0.007**
**Unplanned reintubation**	3 (0.4%)	21 (0.3%)	0.74
**Urinary tract infection**	5 (0.7%)	21 (0.3%)	0.20
**Pneumonia**	1 (0.1%)	13 (0.2%)	> 0.99
**Hematoma**	0 (0%)	8 (0.1%)	> 0.99
**Seizure**	1 (0.1%)	6 (< 0.1%)	0.55
**Venous thromboembolic events**	1 (0.1%)	5 (< 0.1%)	0.50
**Neurological deficits**	0 (0%)	2 (< 0.1%)	> 0.99
**Unspecified complication**	32 (4.3%)	200 (3.2%)	0.15
**Total length of hospital stay (days)**	5.17 (8.32)	3.64 (3.81)	**0.010**
**Extended hospital stay (> 30 days)**	23 (3.1%)	38 (0.6%)	** < 0.001**
**Discharge disposition**			0.075
Home	739 (98%)	6,105 (99%)	
Rehabilitation	7 (0.9%)	34 (0.6%)	
Skilled Care	4 (0.5%)	15 (0.2%)	
Expired	2 (0.3%)	4 (< 0.1%)	
**30-day unplanned readmission**	71 (9.4%)	438 (7.1%)	**0.021**
**Readmission reasons**			
Headache or neurological deficits	22 (31%)	144 (33%)	
Wound-related	21 (30%)	135 (31%)	
Meningitis	4 (5.6%)	33 (7.5%)	
Cerebrospinal fluid leak	10 (14%)	31 (7.1%)	
Intracranial hemorrhage	0 (0%)	11 (2.5%)	
Other infection	5 (7.0%)	27 (6.2%)	
Other	9 (13%)	57 (13%)	
**30-day reoperation**	50 (6.6%)	177 (2.9%)	** < 0.001**
**Reoperation indications**			
Evacuation of fluid/hemorrhage	16 (32%)	65 (37%)	
Shunt placement/revision/removal	14 (28%)	47 (27%)	
Wound revision	13 (26%)	48 (27%)	
Leak-repair	3 (6.0%)	10 (5.6%)	
Repeat surgery	4 (8.0%)	7 (4.0%)	
**Time to reoperation (days)**	15.06 (7.59)	14.14 (7.11)	
**30-day mortality**	2 (0.3%)	3 (< 0.1%)	0.095

The total length of hospital stay was significantly longer for the syndromic group, with a mean of 5.17 days (SD 8.32) compared to 3.64 days (SD 3.81) in the non-syndromic group (*p* = 0.010). The syndromic group also had a higher rate of extended hospital stays, with 3.1% (*n* = 23) staying longer than 30 days, compared to 0.6% (*n* = 38) of the non-syndromic group (*p* < 0.001).

Regarding readmissions, 9.4% (*n* = 71) of the syndromic patients were readmitted within 30 days, vs. 7.1% (n = 438) of the non-syndromic patients (*p* = 0.021). The most common reasons for readmission were headaches and or neurological deficits, followed by wound-related issues, CSF leak, and meningitis. In addition, the syndromic group had a significantly higher rate of 30-day reoperation (6.6%, *n* = 50 vs. 2.9%, *n* = 177; *p* < 0.001), with the most common reasons for reoperation being fluid/hemorrhage evacuation and shunt placement/revision/removal.

30-day mortality was low in both groups, with 0.3% (*n* = 2) in syndromic and less than 0.1% (*n* = 3) in non-syndromic patients, a difference that was not statistically significant (*p* = 0.095).

In the multivariable logistic regression model (Table 5), which was adjusted for all baseline patient and surgical characteristics including but not limited to sex, age, BMI, race, structural pulmonary abnormalities, previous cardiac surgery, ASA class, hydrocephalus, central apnea, syringomyelia, scoliosis, tethered cord, use of arthrodesis, and operation time, it turned out that syndromic presence was not independently associated with an increased risk of 30-day complications (odds ratio [OR] 0.94, 95% confidence interval [CI] 0.71–1.23, *p* = 0.66) or 30-day readmission (OR 0.96, 95% CI 0.71–1.29, *p* = 0.80). However, syndromic status was an independent and significant predictor associated with an increased risk of 30-day reoperation (OR 1.51, 95% CI 1.03–2.19, *p* = 0.032).

**Table 5:** Multivariable logistic regression model for 30-day complication, readmission, and reoperation risk following posterior fossa decompression in patients with and without associated syndromes

Outcome	Odds ratio	95% CI	*p*-value
**30-day complication**			
Presence of syndrome*	0.94	0.71, 1.23	0.66
**30-day readmission**			
Presence of syndrome*	0.96	0.71, 1.29	0.80
**30-day reoperation**			
Presence of syndrome*	1.51	1.03, 2.19	**0.032**

*Adjusted for several covariates including sex, age, BMI, race, ethnicity, structural pulmonary abnormalities, previous cardiac surgery, cognitive status, seizure, cerebral palsy, neuromuscular disorders, steroid use, nutritional support, hematologic disorders, malignancy, admission type, time from admission to surgery, ASA class, hydrocephalus, central apnea, syringomyelia, scoliosis, tethered cord, arthrodesis, and operation time

## Discussion

In this study of a cohort of patients undergoing PFD for posterior fossa crowding, significant differences in baseline characteristics and postoperative outcomes between patients with and without associated syndromes or conditions were observed. These findings support our initial hypothesis that posterior fossa crowding, when linked to an underlying condition, may be considered a distinct entity with unique characteristics and specific surgical outcomes. In these cases, the pathophysiology of the malformation is likely related to the underlying congenital pathology or syndrome, as opposed to being idiopathic in true CM1 patients with no underlying congenital diseases.

### Associated syndromes

In our study, we identified posterior fossa crowding in association with over 20 distinct congenital conditions, many of which have been previously reported in the literature as linked to CM1 or posterior fossa crowding.

### Craniofacial defects

The posterior cranial fossa and craniofacial structures share a common embryological origin, and their proper development depends on coordinated interactions between the paraxial mesoderm and the ectoderm [18]. It is believed that disruptions in the interaction between these embryonic tissues may contribute to the development of both craniofacial anomalies and posterior fossa crowding.

Often associated with various syndromic abnormalities, posterior fossa crowding is often found in conditions such as craniosynostosis and Pierre Robin syndrome [19, 20].

This highlights the potential link between posterior fossa crowding and complex craniofacial syndrome and suggests that patients with craniofacial defects may be at an increased risk for posterior fossa crowding.

This aligns with the findings of Lara-Reyna et al., [21] who described a patient who presented with cleft palate and was later found to have a posterior fossa crowding. Additionally, Hung et al. [22] described a Taiwanese infant with oto-palato-digital syndrome type 2 and posterior fossa crowding, suggesting a developmental abnormality in neural tube closure.

In one study, posterior fossa crowding was found in 8% of craniosynostosis patients that required craniosynostosis repair, and particularly those with lambdoid suture involvement. While some patients improved following craniosynostosis repair alone, others required a subsequent PFD surgery. Interestingly, the authors of this study also observed that posterior fossa crowding developed in some patients years after craniosynostosis repair, suggesting that in these cases, posterior fossa crowding may share a common pathophysiology with the underlying disease, rather than being caused by the craniosynostosis itself [23].

In one report, posterior fossa crowding was described in a patient with Duchenne muscular dystrophy (DMD), who also presented with frontometaphyseal dysplasia, craniosynostosis, and scoliosis [24]. The authors suggested that the condition in this case may have been incidental and unrelated to DMD. Consistent with previous literature [23], posterior fossa crowding in this case was more plausibly attributed to the coexisting craniosynostosis rather than to DMD itself.

### Connective tissue disorders

As reported by Clarke et al., [25] among patients with CM1 undergoing suboccipital decompression, 0,9% had a connective tissue disorder. Of these, Ehlers-Danlos syndrome (EDS) was the most common, (90.4%) followed by Marfan syndrome (7.9%) and osteogenesis imperfecta (OI) (1.7%) [25]. A large cohort study investigated 2,813 patients with CM1 reported that 12.7% had a coexisting hereditary disorder of connective tissue (HDCT) [26]. Among these, 42% were diagnosed with the hypermobile type of Ehlers–Danlos syndrome (HT-EDS), 27% with the classical form of EDS, and 5% with Marfan syndrome. Notably, the study identified a marked female predominance, with 89% of HDCT/CM1 cases occurring in female patients. A prospective study on 21 HT-EDS patients, found one female patient with CM1 that presented with headache, fatigue, and autonomic dysfunction [27]. Similarly, co-occurrence of Marfan syndrome and CM1 has also been previously described [28].

### Cystic fibrosis

Cystic fibrosis (CF) is rarely associated with posterior fossa crowding, but several cases have described coexistence of the two conditions. In two cases, PFD led to symptom resolution, while patients who were not surgically treated showed persistent or worsening symptoms [29, 30]. In another case, spontaneous regression of syringomyelia and tonsillar herniation was observed [31]. A case report described two pediatric patients with known posterior fossa crowding who died and were posthumously diagnosed with CF based on autopsy findings and postmortem genetic analysis. The authors speculated on a potential link between the conditions, suggesting that CFTR mutations may influence brainstem development or that posterior fossa crowding could result from metabolic and electrolyte imbalances characteristic of CF.

### Achondroplasia

In achondroplasia, abnormal endochondral ossification may result in a narrowed foramen magnum and a small posterior fossa, which can predispose individuals to Chiari-like features [32]. One case report described brainstem descent—an uncommon finding in achondroplasia alone— which the authors suggest is due to a combined effect of achondroplasia and posterior fossa crowding [33].

### Neurofibromatosis

In a cohort study by Tubbs et al. of 130 patients that were surgically treated with PFD, 5.4% had a concomitant neurofibromatosis type 1 (NF1) diagnosis. Conversely, CM1 was identified in 8.4% of a separate cohort of 202 patients with NF1 [34]. Given the relatively high overlap between these two conditions, despite the low individual incidence of each, the authors suggested that the co-occurrence is unlikely to be spurious, supporting a potential true association between CM1 and NF1.

### Other congenital conditions

Posterior fossa crowding has also been reported in association with velocardiofacial syndrome, as shown by Hultman et al. [35], identifying it in 21,1% of patients undergoing MRI. One symptomatic patient presenting with occipital headaches underwent suboccipital craniectomy, C1 laminectomy, and PFD, which resulted in resolution of their symptoms.

Several case reports have described posterior fossa crowding in association with other diverse conditions, with some authors suggesting a non-incidental relationship [28, 36–39]. For instance, Germain et al. reported CM1 in 7.6% of 52 patients with Fabry disease during prospective, systematic MRI screening [37]. The authors suggest that chronic hyperperfusion in the posterior circulation, previously observed in Fabry disease, may contribute to the development of CM1. The authors advised considering CM1 in Fabry patients with cerebellar symptoms, as PFD can be needed.

Another case reports on incidentals findings of posterior fossa crowding and syringomyelia in a boy with Hurler syndrome [36] who underwent suboccipital craniectomy and C1 laminectomy, during which a thickened posterior fossa membrane and an arachnoid cyst were identified. The authors propose that glycosaminoglycan accumulation in Hurler syndrome may lead to thickened arachnoid membranes and meningeal changes, contributing to posterior fossa crowding and cerebellar tonsillar herniation, factors that may mimic CM1 in this patient.

Another case involved a female infant with genetically confirmed Leigh syndrome, where imaging revealed posterior fossa crowding and hydrocephalus requiring CSF drainage. In their study, the authors proposed that posterior fossa crowding may share a similar pathophysiology as the underlying congenital condition itself, referencing similar malformations seen in other metabolic disorders with lactic acidosis [38].

In Klippel-Feil syndrome, posterior fossa crowding co-occurred with hydrocephalus and required PFD surgery [40]. The association between Klippel-Feil syndrome and CM1 has been highlighted in other studies [34].

In a patient with Klinefelter syndrome and a coexisting cerebral germinoma, posterior fossa crowding was identified incidentally on postoperative imaging [41]. Another case described posterior fossa crowding in the setting of pituitary dwarfism with associated syringomyelia [42].

Multiple reports have documented an association between posterior fossa crowding and fetal alcohol spectrum (FAS). One case described by Nicita et al. [43] involved a patient with focal motor seizures, syringomyelia, and microcephaly, but only mild developmental delay. Another report detailed a patient with FAS and posterior fossa crowding who presented with significant intellectual and neuropsychiatric impairments [44]. Although the underlying mechanism remains unclear, preclinical studies suggest that prenatal alcohol exposure can disrupt normal craniofacial development, affecting the neurocranium even more significantly than the viscerocranium [45].

Other rare associations include Prader-Willi and Angelman syndromes, in which posterior fossa crowding has been occasionally reported but is likely underdiagnosed due to the lack of routine MRI imaging in their standard diagnostic workup [18, 46].

### Difference in baseline characteristics

In terms of baseline characteristics, syndromic patients were found to have significantly higher rates of various comorbidities and exhibited higher ASA scores. This was expected, since underlying syndromes are commonly accompanied with a wide array of comorbidities affecting multiple organ systems.

In our study, syndromic posterior fossa crowding was more frequently associated with hydrocephalus and central sleep apnea compared to the non-syndromic group. Numerous prior studies and case reports support the strong association between hydrocephalus and posterior fossa crowding in syndromic contexts, including achondroplasia [33], craniosynostosis [23], Klippel-Feil syndrome [40], Leigh syndrome [38], Angelman syndrome [46], and cystic fibrosis [39].

Similarly, our observed increase rate of central sleep apnea among syndromic cases aligns with previous findings, including studies on patients with concomitant neurofibromatosis type 1 [34] and velocardiofacial syndrome [35].

Conversely, the rates of scoliosis, syringomyelia, and tethered cord appeared similar across both groups in our study population, which contrasts with previous studies and case reports which support a potential overrepresentation of the aforementioned features among syndromic cases [34, 47, 48].

Syringomyelia was commonly reported in association with syndromic cases, notably in patients with neurofibromatosis type 1 [34], cystic fibrosis [31], pituitary dwarfism [42], and Hurler syndrome [36].

### Surgical outcomes

Based on our findings, syndromic patients were more likely to experience complications, readmissions, and reoperations within 30 days following PFD surgery. However, after adjusting for multiple potential confounders, the presence of associated syndromes was not independently linked to an increased risk of postoperative complications or readmissions. Notably, syndromic status was only identified as an independent risk factor for reoperation within 30 days. This suggests that the higher rates of complications and readmissions in the syndromic group were influenced by other confounding variables, emphasizing that the presence of an underlying syndrome alone should not deter surgical treatment of these patients.

Differences in the surgical management of syndromic versus non-syndromic patients with posterior fossa crowding have been described by Saddler et al., [49] who reported syndromic patients were diagnosed earlier and more commonly underwent PFD, which the authors attributed to earlier and more extensive imaging as well as more severe disease presentations. Provenzano et al. [50] found no significant differences in surgical outcomes between syndromic and non-syndromic patients, nor between the various underlying syndromes. Greenberg et al. [51] initially observed that congenital and genetic comorbidities were associated with higher postoperative complication rates. However, after adjusting for confounding variables in a multivariate analysis, this association was no longer significant—only hydrocephalus remained an independent predictor of postoperative complications. This echoes the finding from previous studies, where hydrocephalus emerged as the most prominent predictor of outcomes following PFD [52, 53].

### Limitations

This study has several limitations, primarily related to the use of the NSQIP database. First, the dataset contains missing or incomplete information, particularly regarding syndromic diagnoses, leading to a potentially significant number of unidentified syndromic cases. Additionally, certain conditions commonly associated with CM1 and posterior fossa crowding, such as scoliosis, hydrocephalus, and syringomyelia, may be underreported due to the nature of coding and data collection within the database. The NSQIP Pediatric database does not provide sufficiently granular data to specify the exact surgical technique performed. Therefore, we are unable to stratify outcomes based on these specific approaches. The lack of patient-reported outcome measures limits the ability to assess functional or quality-of-life outcomes following surgery. Most importantly, the syndromic group included in this study is heterogeneous, encompassing a range of genetic conditions with potentially distinct pathophysiological mechanisms contributing to posterior fossa crowding. This variability should be considered when interpreting and generalizing the findings.

## Conclusion

Syndromic posterior fossa crowding is associated with distinct baseline characteristics and features, as well as a higher rate of short-term adverse events following PFD compared to true CM1. Although syndromic patients witnessed higher rates of overall complications, readmissions, and reoperations, syndromic status only acted as an independent predictor of reoperation, but not complications or readmissions. This suggests that syndromic status alone should not be a deterrent to surgery, and that surgical decisions should instead focus on the patient’s specific comorbidities rather than the presence of a syndrome itself. Finally, findings from this study suggest syndromic Chiari-like posterior fossa crowding represents a clinically and etiologically distinct entity, warranting tailored management and further investigation.

## Data Availability

No datasets were generated or analysed during the current study.
